# Correlates of psychological resilience and risk: Prospective associations of self‐reported and relative resilience with Connor‐Davidson resilience scale, heart rate variability, and mental health indices

**DOI:** 10.1002/brb3.2091

**Published:** 2021-02-27

**Authors:** Sun Jae Jung, Ye Jin Jeon, Karmel W. Choi, Ji Su Yang, Jeong‐Ho Chae, Karestan C. Koenen, Hyeon Chang Kim

**Affiliations:** ^1^ Department of Preventive Medicine Yonsei University College of Medicine Seoul South Korea; ^2^ Department of Public Health Yonsei University Graduate School Seoul South Korea; ^3^ Department of Epidemiology Harvard T.H. Chan School of Public Health Boston MA USA; ^4^ Department of Psychiatry Massachusetts General Hospital Boston MA USA; ^5^ Department of Psychiatry St. Mary’s Hospital Seoul South Korea

**Keywords:** CD‐RISC, depression, heart rate variability, loneliness, longitudinal study, resilience

## Abstract

**Background:**

There are several ways to determine psychological resilience. However, the correlation between each measurement is not clear. We explored associations of baseline relative “resilience” and risk with later self‐reported trait resilience and other biological/mental health indices.

**Methods:**

We utilized baseline and follow‐up survey data from 500 participants aged 30–64 in the community cohort. Baseline “relative” resilience was defined by: (a) negative life events (NLEs) in the six months before baseline and (b) depressive symptoms at baseline, yielding four groups of individuals: i) “Unexposed and well,” “Vulnerable (depression),” “Reactive (depression),” and “Resilient.” “Trait” resilience at follow‐up was self‐reported using the Connor‐Davidson Resilience Scale (CD‐RISC). Associations between relative resilience at baseline, CD‐RISC, and heart rate variability (HRV) indices at follow‐up were assessed with generalized linear regression models after adjustments. Associations between baseline resilience and subsequent loneliness/depression indices were also evaluated.

**Results:**

Overall trait resilience and its subfactors at follow‐up showed strong negative associations with “Reactive” at baseline (adj‐β for total CD‐RISC score: −11.204 (men), −9.472 (women)). However, resilience at baseline was not associated with later HRV, which was compared with the significant positive association observed between CD‐RISC and HRV at the same follow‐up time point. The “Reactive” exhibited significantly increased depressive symptoms at follow‐up. The overall distribution pattern of CD‐RISC subfactors differed by baseline resilience status by sex.

**Conclusions:**

The “relative” resilience based on the absence of depression despite prior adversity seems to be highly related with trait resilience at follow‐up but not with HRV. The sub‐factor pattern of CD‐RISC was different by sex.

## Graphical abstract

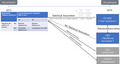

## 1 INTRODUCTION

Psychological resilience is multidimensional and can be defined in various ways. For example, a new research agenda for resilience research gives working definitions of resilience as a (a) capacity (or trait), (b) process (or adaptivity to stressful/traumatic event), and (c) outcome (Choi et al., 2019). Various measures have been applied to assess resilience. As trait resilience can be interpreted as a more distal and lasting characteristic, state resilience is construed as more recent and responsive to life events. Some studies have integrated these two concepts, creating the State‐Trait Assessment of Resilience Scale (STARS) to reflect both trait and state resilience (Lock et al., 2020). Several studies have operationally defined resilience in various ways, such as the absence of time lost due to illness after psychological stress or a lack of lifetime psychiatric disorders after exposure to traumatic life events (Amstadter et al., 2014; Yehuda et al., 2006). Others administered structured scales (Bartone et al., 1989; Wagnild & Young, 1993), among which the Connor‐Davidson Resilience Scale (CD‐RISC; Connor & Davidson, 2003) is the most frequently used in investigating resilience traits. CD‐RISC is also well known to reflect the biological aspects of resilience (An et al., 2019; Connor & Davidson, 2003). However, results comparing the measurement trait of CD‐RISC and other biological markers are insufficient. Power spectrum analysis of heart rate variability (HRV) has been suggested as one global index of psychophysiological resilience; this measurement is known to reflect sympathovagal balance related to autonomic flexibility (An et al., 2019). Additionally, some studies report psychological resilience was associated with stress reactivity measures such as hair cortisol and hypotalamic–pituitary–adrenal axis reactivity including cardiovascular and electrodermal measurement of heart rate and skin conductance level (Lehrer et al., 2020; Winslow et al., 2015). Comparing these measurement modalities may aid in disentangling the complexity of resilience.

Additionally, it is essential to recognize that the term “resilience” implies both cross‐sectional and temporal aspects: trait resilience and the relative or outcome‐based resilience. To clarify, both assessments should be made longitudinally and compared. The consistency between an operationally defined “relative” resilience state definition and the later measurement of “trait or state” resilience measured with CD‐RISC should be examined to assess the multidimensionality of this complicated term. A comparison of “state” resilience markers, including CD‐RISC and HRV, and operationally defined “relative” resilience would indicate whether relative resilience is linked to later trait resilience.

A number of studies have evaluated resilience state as a predictor of other mental health outcomes, especially depression (Hjemdal et al., 2007; Min et al., 2013). Although the heterogeneity of depression is widely recognized (Goldberg, 2011), it is still unknown whether resilience is protective, and particularly for which aspect of depression subtype. Loneliness is another outcome for which the impact of resilience has been examined; however, the results are not consistent regarding the association between resilience and loneliness (Gerino et al., 2017; Perron et al., 2014).

Additionally, a number of previous literatures have evaluated the gender difference in resilience; some research reported that women are more vulnerable in the aspect of psychological resilience (Bonanno et al., 2011), and this deviation was suggested as the result from different social‐ecological stressors, social support and resources, and power to negotiate between men and women (Riger, 2001). Furthermore, gender difference was reported regarding the multidimentional nature of psychological resilience; one study, including people who experience spousal loss, suggested that gender influenced each sub‐dimensions (i.e., life satisfaction, negative affect, and positive affect) of resilience differently (Infurna & Luthar, 2017), and a longitudinal study from elderly population asserted that there was different gender effect regarding the association between each resilience subdomain (e.g., physical activity, emotional support, and solitary leisure activity) and mortality. (Walter‐Ginzburg et al., 2005).

In this study, we compared baseline “relative” resilience and risk, defined operationally by the presence of negative event/consequent depression, with “trait” resilience measured with CD‐RISC and HRV approximately five years later in each gender. Furthermore, we compared baseline resilience and risk with other mental health outcomes, loneliness, and depressive symptom at follow‐up.

## 2 METHODS

### 2.1 Selection of the participants

We used data from the 1st follow‐up of the Cardiovascular and Metabolic Disease Etiology Research Center (CMERC) cohort, an ongoing prospective population study. A total of 11,964 participants completed the baseline survey from 2013 to 2018 [12]. Of the 807 participants who were enrolled in 2013, we excluded people (a) consuming excess alcohol (men ≥ 30 g/day, women ≥ 20 g/day), (b) with chronic hepatitis virus infection history, (c) having a history of malignant tumor, (d) missing data on laboratory assessment or image data, (e) pregnant or lactating, (f) participating in any clinical trial, and (g) unable to read the informed consent form, leaving 500 participants in the follow‐up survey in 2019. Compared with the excluded people, participants who were included in the final analysis were older, more menopaused in women, and less depressed. (Table S1.)

### 2.2 Measurements

In the baseline assessment, participants of the CMERC cohort provided information about their sociodemographic variables, physical status including disease history, lifestyle factors, stressful life events in the past six months, and depression status measured with the Beck Depression Inventory (BDI)‐II, and their height and weight were measured. To measure stressful life events, the first section of the Life Experience Survey (LES; Sarason et al., 1978), with 47 items targeting the general adult population, was used. Participants were asked whether they had experienced the listed stressful events, such as the death of a close family member or problems in their workplaces, in the past six months. If the respondents reported any items from the questionnaire, an additional question on the impact of the corresponding event was given as a Likert scale; the influence of the item could be rated from −3 (*extremely negative*) to +3 (*extremely positive*).

In the follow‐up survey of 2019, most of the measurements were repeated from the baseline assessment. Trained interviewers covered all items in the questionnaire. Compared with the baseline survey, several additional measurements were made at the follow‐up. In short, trained interviewers administered the Connor‐Davidson Resilience Scale (CD‐RISC) and UCLA Loneliness Scale (ULS), and heart rate variability (HRV) was measured with SA‐2000E (Medicore Co.). The 25‐item CD‐RISC is scored on a 5‐point Likert scale (0–4), where a higher score reflects greater trait resilience. This scale has been validated in hospitals and the general population, showing high convergent validity, adequate internal consistency (Cronbach's *α* = .92), and test–retest reliability (*r* = .875); Jung et al. also suggested five subfactors after confirmatory factor analysis, using the same validation population (Jung et al., 2012). Heart rate variability reflects the autonomic input to heart rate, allowing an estimation of the transition of autonomic tone (Stein et al., 1994). Higher‐frequency (HF) power is reported as a marker of vagal influence, whereas low‐frequency (LF) power is a marker of cardiac sympathetic tone and parasympathetic modulation (Tsuji et al., 1994). In the morning after breakfast time, participants had a 5‐min preparation time, sitting in a relaxed way. The HRV device has three electrocardiogram sensors applied on each participant's right/left wrist and left ankle. Electrocardiogram data were collected at a rate of 500/s for 5 min, followed by HRV data analysis. Low HRV has been reported to be associated with adverse mental health events such as anxiety or depression. Reduced HF and LF/HF ratio are known to associate post‐traumatic stress disorder symptoms (An et al., 2020). The standard deviation of the NN interval (SDNN) is an index highly correlated with the sympathetic and parasympathetic nervous system. The physical stress index (Psi) reflects the pressure given on the regulation system. Total power includes HF, LF, and very low frequency, which reflect the autonomic nervous system's overall activity. Root mean square of differences between successive NN intervals (RMSSD) is used as an index of parasympathetic outflow (An et al., 2020; Shaffer & Ginsberg, 2017). The screening tool for depressive symptoms at follow‐up was the Patient Health Questionnaire‐9 chosen to replace the BDI‐II for greater brevity of the overall questionnaire.

### 2.3 Defining psychological resilience at baseline and follow‐up

For the baseline relative resilience definition, we adopted the criteria from our previous study (Jung et al., 2020). When a participant gave a negative score (−1 to −3) for the impact of any item, we counted it as a “negative life event (NLE).” Participants were grouped into four categories based on the presence of NLE and depressive symptoms, the latter defined by a BDI‐II score of 20 or higher, (Dozois et al., 1998) yielding four categories: (a) “Unexposed and well” (no NLE with no depressive symptom), (b) “Resilient” (with NLE but no symptom), (c) “Reactive” (with NLE and with symptom), and (d) “Vulnerable” (no NLE but with symptom).

For the analysis of CD‐RISC measured at follow‐up, we applied the five‐factor structure found by the original validation study (Jung et al., 2012) to our current data: factor 1 for the “driving force for achievement” (items 6, 10, 21, 22, 23, 24, and 25), factor 2 for “adaptability to adversity or stressful situations” (items 8, 12, 13, 14, 16, 17, and 19), factor 3 for the “resource to overcome adversity” (items 1, 2, 4, 5, 11, and 13), factor 4 for “self‐direction” (items 15, 18, and 20), and factor 5 for “conformity to destiny” (items 3 and 9).

### 2.4 Covariates

The demographic variables at the baseline were educational level, household income, and marital status. The final educational level was categorized as elementary, middle, high school, university or college, or above. The average monthly income of the family was assessed as quartiles on a cumulative distribution. Marital status was treated as a categorical variable as “living together with a partner,” “divorced,” “widowed,” “never married or cohabited,” and “other.” Comorbidity was defined as a history of any of the listed diseases diagnosed by physicians as follows: hypertension, diabetes, any cancer, stroke and transient ischemic stroke, myocardial infarct and angina, heart failure, chronic renal failure, dyslipidemia, liver diseases including fatty liver disease, chronic hepatitis, liver cirrhosis, thyroid disorders, asthma, chronic obstructive pulmonary disease, osteoporosis, arthritis, and autoimmune disease. Body mass index was calculated as dividing weight by squared height (kg/m^2^). Lifestyle variables such as cigarette smoking and alcohol consumption were also categorized as current, past, and never smoker/user. For the physical exercise variable, the “physically active group” was defined following the World Health Organization guidelines (World_Health_Organization, 2010) as people with at least 150 min of moderate or 75 min of vigorous aerobic activity during the week on average. For women, menopausal status was categorized as “menopause,” “perimenopause,” and “pre‐menopause.”

### 2.5 Statistical analysis

The four groups defined by baseline relative resilience status were compared with various demographic, physical, and lifestyle variables, using ANOVA for continuous variables and chi‐square tests for the categorical variables.

To estimate the associations between baseline operationally defined resilience status and CD‐RISC scores at follow‐up, including the total and subscores by factor, a generalized linear mixed model was used with “Resilient” as a reference group, after adjusting for demographic factors, lifestyle factors, comorbidity, and menopausal status in the case of women. The same method was used to assess baseline resilience status and CD‐RISC with indices from HRV measurement at follow‐up. For the multiple comparison, we applied Bonferroni adjustment (0.05/6). Additionally, a radar chart with five axes for the standardized sub‐factor scores of the CD‐RISC at follow‐up was plotted with the standardized scores by baseline resilience status for each sex.

Associations between baseline resilience status and other mental indices at follow‐up were also analyzed using the generalized linear mixed model. For PHQ‐9 at follow‐up, we applied a two‐factor structure, “cognitive‐affective” depression and “somatic‐affective” depression, from our previous analysis. Cognitive depression comprised items 1–5 from the PHQ‐9, including anhedonia and hopelessness, whereas somatic depression comprised PHQ‐9 items 6–9, suggesting change of appetite, psychomotor retardation, and difficulty in concentrating (Lee et al., 2020). Furthermore, we examined associations between the two measurements at follow‐up of the CD‐RISC score and heart rate variability indices using the same generalized linear mixed model.

### 2.6 Statement of ethics

The protocol of this study was approved by the Institutional Review Board of Yonsei University (YUIRB‐ 4‐2013‐0661), and written informed consent was provided by all participants. All procedures in this work complied with the ethical standards of the relevant national and institutional committees on human experimentation and with the Helsinki Declaration of 1975, as revised in 2008.

## 3 RESULTS

The four groups defined by resilience at the initial assessment showed an overall difference in age and cigarette smoking. The “Resilient” at baseline tended to be younger and to smoke less. The “Vulnerable” tended to be older and to smoke more than the other groups. However, other variables such as family income, marital status, comorbidity, alcohol consumption, exercise, and menopausal status in women did not show any significant difference at the baseline (Table 1). The mean scores of the CD‐RISC were 69.72 (*SD* = 14.1) in men and 68.14 (*SD* = 16.4) in women, which did not significantly differ (*p* = .276).

**TABLE 1:** Baseline characteristics in CMERC participants by operational definition of Resilience status at initial assessment (*N* = 500)

Participants' characteristics	Unexposed and well^a^ (*N* = 168)	Resilient^b^ (*N* = 280)	Reactive Depression^c^ (*N* = 42)	Vulnerable Depression^d^ (*N* = 10)	*p*‐value
Age, Mean (*SD*)	53.13 (7.3)	50.66 (9.2)	53.93 (6.9)	54.70 (5.3)	.004
Female, *N* (%)	113 (67.3)	195 (69.6)	33 (78.6)	8 (80.0)	.468
Socioeconomic variables					
Education: Highschool or more, *N* (%)^f^	61 (36.3)	122 (43.6)	12 (28.6)	2 (20.0)	.096
Highest quartile of yearly Household income, *N* (%)^f^	34 (20.2)	47 (16.8)	5 (11.9)	2 (20.0)	.591
Currently married, living together, *N* (%)	150 (89.3)	242 (86.4)	36 (85.7)	7 (70.0)	.324
Presence of major comorbidity, *N* (%)^f^	85 (50.6)	124 (44.3)	23 (57.8)	5 (50.0)	.437
Hypertension, *N* (%)^f^	41 (24.4)	58 (20.7)	10 (23.8)	3 (30.0)	.747
Diabetes, *N* (%)^f^	19 (11.3)	31 (11.1)	6 (14.3)	0 (0)	.644
Body mass index (kg/m^2^), Mean (*SD*)	23.4 (2.8)	23.9 (2.8)	23.5 (3.1)	25.4 (4.0)	.064
Lifestyle factors, *N* (%)					
Current cigarette smoker^f^	11 (6.6)	30 (10.7)	6 (14.3)	3 (30.0)	.053
Current alcohol consumer	98 (58.3)	171 (61.1)	24 (57.1)	8 (80.0)	.545
Regular exercise^e^, ^f^	76 (45.2)	146 (52.1)	16 (38.1)	3 (30.0)	.146
Menopaused (women only)	83 (73.5)	120 (61.5)	24 (72.7)	6 (75.0)	.142
Psychiatric assessments					
Beck Depression Inventory II (range:0–63)	6.4 (4.5)	9.1 (4.9)	26.0 (4.7)	22.9 (3.0)	<.001
Mini Mental State Examination‐DS (range: 0–30)	27.4 (1.6)	27.3 (1.8)	26.9 (2.3)	25.8 (2.0)	.038

^a^ No negative event experience in 6 months and no current depressive symptoms (BDI < 20).

^b^ Experienced negative events in 6 months but no current depressive symptoms (BDI < 20).

^c^ Experienced negative events in 6 months with current depressive symptoms (BDI ≥ 20).

^d^ No negative event experience in 6 months and with current depressive symptoms (BDI ≥ 20).

^e^ Defined as having moderate‐vigorous physical activity more than 150 min in a week in average.

^f^ Fisher's exact Test.

The total CD‐RISC score at follow‐up showed significant negative associations with the baseline “Reactive” group in both men (adjusted‐*β* = −11.204, *p* = .025) and women (adjusted‐*β* = −9.472, *p* = .002) compared with the “Resilient.” This pattern remained for all subfactors in both sexes. The baseline “Vulnerable” group also showed negative associations with CD‐RISC and its subfactors at follow‐up, though without statistical significance (Table 2).

**TABLE 2:** Association between resilience operationally defined at baseline and Connor‐Davidson Risk Score measured at follow‐up

Connor‐Davidson Resilience Scale at follow‐up	Relative Resilience State at baseline									
	Unexposed and well^a^ (*N* = 168)			Resilient^b^ (Ref.) (*N* = 280)	Reactive depression^c^ (*N* = 42)			Vulnerable depression^d^ (*N* = 10)		
	Mean (*SD*)	Adj.‐β^e^ (*SE*)	*p*‐value	Mean (*SD*)	Mean (*SD*)	Adj.‐β^e^ (*SE*)	*p*‐value	Mean (*SD*)	Adj.‐β^e^ (*SE*)	*p*‐value
Men, *N*		55		85		9			2	
Total score	73.2 (12.7)	3.376 (2.49)	.178	69.0 (14.6)	57.0 (11.5)	**−11.204 (4.93)**	**.025**	62.0 (2.8)	−10.263 (9.88)	.301
Factor 1: Driving force for achievement	20.9 (4.2)	1.439 (0.82)	.082	19.4 (4.8)	16.1 (3.6)	**−2.822 (1.64)**	**.088**	18.5 (0.7)	−1.542 (3.29)	.640
Factor 2: Adaptability to adversity/stressful situation	20.7 (4.0)	0.887 (0.80)	.268	19.6 (4.7)	15.8 (4.0)	**−3.857 (1.60)**	**.017**	17.5 (0.7)	−3.372 (3.20)	.294
Factor 3: Resource to overcome adversity	18.1 (3.6)	0.801 (0.68)	.244	17.3 (4.1)	13.3 (3.4)	**−3.564 (1.37)**	**.010**	12.5 (3.5)	**−5.550 (2.75)**	**.045**
Factor 4: Self‐direction	8.6 (1.8)	0.728 (0.33)	.027	7.8 (1.9)	6.9 (2.1)	**−0.807 (0.65)**	**.216**	7.5 (0.7)	−0.810 (1.30)	.535
Factor 5: Conformity to destiny	5.0 (1.4)	0.001 (0.27)	.996	4.9 (1.6)	3.9 (1.1)	−1.068 (0.54)	.051	5.0 (1.4)	−0.029 (1.09)	.979
Women, *N*		113		195		33			8	
Total score	70.5 (17.4)	1.702 (1.93)	.379	68.7 (15.3)	57.9 (16.0)	**−9.472 (3.09)**	**.002**	63.4 (14.8)	−3.467 (6.46)	.592
Factor 1: Driving force for achievement	19.5 (5.4)	0.246 (0.64)	.702	19.1 (5.1)	16.0 (6.2)	**−3.000 (1.03)**	**.004**	16.3 (4.7)	−2.754 (2.00)	.17
Factor 2: Adaptability to adversity/stressful situation	20.2 (5.3)	0.878 (0.59)	.136	19.2 (4.7)	16.0 (4.8)	**−2.842 (0.94)**	**.003**	17.4 (6.0)	−1.438 (1.83)	.433
Factor 3: Resource to overcome adversity	18.0 (4.5)	0.728 (0.50)	.147	17.5 (3.9)	15.2 (4.2)	**−1.743 (0.80)**	**.030**	15.3 (6.8)	−1.422 (1.56)	.363
Factor 4: Self‐direction	7.8 (2.3)	0.252 (0.26)	.342	7.6 (2.1)	6.3 (2.3)	**−1.146 (0.42)**	**.007**	7.3 (2.7)	−0.109 (0.82)	.895
Factor 5: Conformity to destiny	5.2 (1.9)	−0.228 (0.21)	.277	5.5 (1.7)	4.7 (1.8)	−0.592 (0.33)	.078	4.0 (1.7)	−1.005 (0.65)	.124

^a^ No negative event experience in 6 months before initial assessment and no depressive symptoms (BDI < 20) at baseline.

^b^ Experienced negative events in prior 6 months before initial assessment but no depressive symptoms (BDI < 20) at baseline.

^c^ Experienced negative events in 6 months before initial assessment with depressive symptoms (BDI ≥ 20) at baseline.

^d^ No negative event experience in 6 months before initial assessment and with depressive symptoms (BDI ≥ 20) at baseline.

^e^ Adjusted for age, study center, education, income, marital status, comorbidity, menopausal status (in women only), alcohol consumption, cigarette smoking, and physical activity.

Contrariwise, we found no significant associations between any resilience‐related category at baseline and the indices of heart rate variability at follow‐up (Table 3). However, when we compared the indicators within the same period, assessing the association between CD‐RISC score and HRV indices at follow‐up, we found significant associations between the CD‐RISC and certain indices of HRV. The total CD‐RISC score showed a statistically significant association with the low‐to‐high frequency ratio (LF/HF) in men (adj‐*β* = 0.052, *p* = .021), and this pattern held as well for the sub‐scores for factors 2 and 3. In women, the total CD‐RISC score showed a positive association only with low frequency (adj‐β = 1.706, *p* = .026), a pattern that also held for factors 1 and 3 (Table S2).

**TABLE 3:** Association between operationally defined resilience status and heart rate variability indices

Heart Rate Variability Indices at follow‐up^a^	Operational definition at baseline										
	Unexposed and well^b^ (*N* = 168)			Resilient^c^ (ref; *N* = 280)		Reactive Depression^d^ (*N* = 42)			Vulnerable Depression^e^ (*N* = 10)		
	Mean (*SD*)	Adj.‐β^a^ (*SE*)	*p*‐value	Mean (*SD*)	Adj.‐β^a^ (*SE*)	Mean (*SD*)	Adj.‐β^a^ (*SE*)	*p*‐value	Mean (*SD*)	Adj.‐β^a^ (*SE*)	*p*‐value
Men, *N*	53			82		9			0		
SDNN	31.2 (15.7)	3.15 (3.08)	.308	31.5 (17.9)	Ref.	27.9 (7.5)	0.04 (6.02)	.995	NA		
Psi	102.2 (124.7)	−5.16 (19.95)	.796	92.7 (107.0)	Ref.	77.3 (58.3)	−40.64 (39.01)	.300	NA		
TP	831.0 (799.0)	137.37 (188.47)	.467	930.2 (1,194.7)	Ref.	583.8 (254.1)	−106.20 (368.61)	.774	NA		
LF	211.5 (274.4)	38.91 (55.74)	.486	240.3 (340.3)	Ref.	164.8 (108.3)	22.46 (109.02)	.837	NA		
HF	147.2 (289.1)	45.08 (41.16)	.276	135.2 (164.4)	Ref.	81.4 (109.4)	−30.55 (80.50)	.705	NA		
LF/HF	3.23 (3.9)	0.46 (0.69)	.502	2.70 (3.9)	Ref.	3.7 (2.9)	1.34 (1.35)	.324	NA		
RMSSD	22.9 (21.8)	2.93 (3.65)	.424	21.9 (18.1)	Ref.	17.4 (7.9)	−3.74 (7.14)	.602	NA		
Women, *N*	112			184		31			8		
SDNN	29.5 (13.2)	−0.32 (1.95)	.868	30.3 (17.8)	Ref.	27.9 (9.5)	−2.66 (3.15)	.3993	23.7 (8.1)	−6.30 (6.00)	.294
Psi	88.8 (80.7)	4.47 (9.77)	.647	83.4 (79.6)	Ref.	86.4 (68.7)	2.36 (15.80)	.8815	113.8 (71.0)	33.95 (30.08)	.260
TP	755.7 (704.6)	−71.05 (190.24)	.709	837.1 (1962.0)	Ref.	640.2 (438.4)	−244.02 (307.87)	.429	433.8 (324.9)	−404.82 (585.97)	.490
LF	187.0 (247.5	29.66 (27.99)	.290	162.8 (229.4)	Ref.	111.6 (95.4)	−41.52 (45.29)	.360	78.5 (45.4)	−63.86 (86.20)	.459
HF	147.4 (190.4)	−28.37 (41.13)	.491	179.9 (416.6)	Ref.	97.6 (99.3)	−95.77 (66.56)	.151	59.6 (36.4)	−124.91 (126.69)	.325
LF/HF	1.9 (2.1)	0.35 (0.23)	.141	1.7 (1.8)	Ref.	2.1 (2.0)	0.66 (0.38)	.082	1.6 (1.0)	0.44 (0.72)	.546
RMSSD	21.6 (13.0)	−2.86 (2.44)	.242	24.4 (24.1)	Ref.	19.6 (9.8)	−6.09 (3.95)	.124	15.1 (5.3)	−9.68 (7.51)	.198

^a^ SDNN, Standard Deviation of the NN interval; Psi, Physical Stress Index or Pressure Index; TP, Total power; LF, Low frequency; HF, High frequency; RMSSD, Square root of the mean of the sum of the square of differences between adjacent NN intervals.

^b^ No negative event experience in 6 months before initial assessment and no depressive symptoms (BDI < 20) at baseline.

^c^ Experienced negative events in prior 6 months before initial assessment but no depressive symptoms (BDI < 20) at baseline.

^d^ Experienced negative events in 6 months before initial assessment with depressive symptoms (BDI ≥ 20) at baseline.

^e^ No negative event experience in 6 months before initial assessment and with depressive symptoms (BDI ≥ 20) at baseline.

**FIGURE 1:**
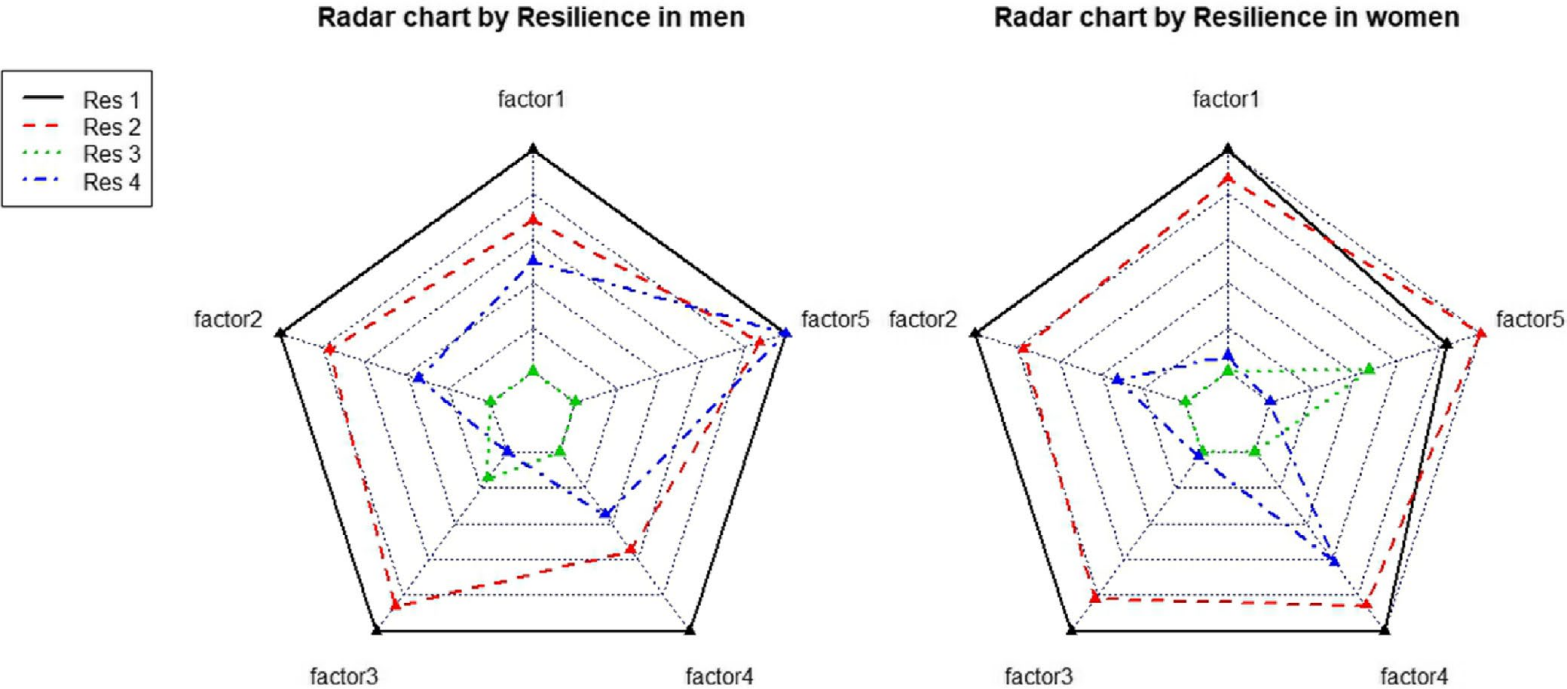
The five subfactors from Connor Davidson Resilience Scale

In the radar chart with the five axes of CD‐RISC subfactors at follow‐up, the overall distribution pattern differed by baseline resilience status between men and women. “Resilient” women showed higher scores overall for the five subfactors of CD‐RISC at follow‐up, whereas “Resilient” men showed relatively low self‐direction. Also, men with “Reactive” depression showed relatively higher resources to overcome adversity, whereas women in the corresponding group showed a higher tendency of conformity to destiny. Conversely, in people with “Vulnerable” depression at baseline, men showed a higher tendency to follow destiny, whereas women showed higher self‐direction (Figure 1).

Comparison of baseline relative resilience and risk categories with other mental health indices at follow‐up found that “Reactive” women and “Vulnerable” men showed strong positive associations with loneliness as measured with the UCLA Loneliness Scale compared with the reference Resilient category (Reactive women: adj‐*β* = 4.63, *p* = .001; Vulnerable men: adj‐*β* = 14.50, *p* = .010). The Reactive group also showed significant positive associations with PHQ‐9 at follow‐up in both men and women (men: adj‐*β* = 2.32, *p* = .043; women: adj‐*β* = 2.87, *p* < .001), exhibiting relatively strong associations with somatic‐affective factor scores (men: adj‐*β* = 1.60, *p* = .025; women: adj‐*β* = 1.87, *p* < .001). In contrast, the “Vulnerable” group did not show a significant association with overall PHQ‐9 at follow‐up. People who were unexposed to adverse events and had no depressive symptoms showed lower associations with loneliness and depression at follow‐up than the “Resilient” (Table 4).

**TABLE 4:** Association between resilience operationally defined at baseline and other psychologic outcomes at follow‐up including Loneliness and Depression

Mental health indices at follow‐up	Operational definition at baseline									
	Unexposed and well^a^			Resilient^b^ (Ref.)	Reactive Depression^c^			Vulnerable Depression^d^		
	(*N* = 168, *M* = 55, *F* = 113))			(*N* = 280; *M* = 85, *F* = 194)	(*N* = 42; *M* = 9, *F* = 33)			(*N* = 10; *M* = 2, *F* = 8)		
	Mean (*SD*)	Adj.‐β^e^ (*SE*)	*p*‐value	Mean (*SD*)	Mean (*SD*)	Adj.‐β^e^ (*SE*)	*p*‐value	Mean (*SD*)	Adj.‐β^e^ (*SE*)	*p*‐value
Men										
UCLA Loneliness Scale	35.9 (8.5)	−0.94 (1.38)	.496	36.0 (7.8)	41.9 (8.5)	3.22 (2.75)	.244	50.5 (2.1)	**14.50 (5.52)**	**.010**
Patient Health Questionnaire−9	1.9 (3.6)	−0.83 (0.57)	.145	2.9 (2.6)	5.1 (5.5)	**2.32 (1.14)**	**.043**	6.5 (2.1)	4.07 (2.28)	.077
Factor1^f^, ^g^: Somatic‐affective	6.1 (2.0)	−0.56 (0.35)	.115	6.8 (1.8)	8.3 (3.4)	**1.60 (0.70)**	**.025**	8.5 (0.7)	1.90 (1.41)	.181
Factor2^f^, ^h^: Cognitive‐affective	4.8 (1.7)	−0.28 (0.26)	.284	5.1 (1.1)	5.8 (2.2)	0.72 (0.51)	.160	7.0 (1.4)	**2.17 (1.03)**	**.037**
Women										
UCLA Loneliness Scale	34.9 (7.1)	−0.95 (0.90)	.290	35.6 (7.6)	41.2 (7.9)	**4.63 (1.44)**	**.001**	42.6 (6.8)	5.19 (2.80)	.065
Patient Health Questionnaire−9	2.2 (2.4)	**−1.18 (0.36)**	**.001**	3.2 (3.3)	6.5 (3.3)	**2.87 (0.58)**	**<.001**	6.0 (2.4)	1.53 (1.12)	.175
Factor1^f^, ^g^: Somatic‐affective	6.4 (1.5)	**−0.69 (0.24)**	**.004**	7.0 (2.2)	9.1 (2.0)	**1.87 (0.38)**	**<.001**	8.3 (1.5)	0.53 (0.74)	.479
Factor2^f^, ^h^: Cognitive‐affective	4.8 (1.1)	−0.49 (0.16)	.003	5.2 (1.4)	6.4 (1.7)	**1.00 (0.26)**	**<.001**	6.8 (1.7)	**1.00 (0.51)**	**.052**

^a^ No negative event experience in 6 months before initial assessment and no depressive symptoms (BDI < 20) at baseline.

^b^ Experienced negative events in prior 6 months before initial assessment but no depressive symptoms (BDI < 20) at baseline.

^c^ Experienced negative events in 6 months before initial assessment with depressive symptoms (BDI ≥ 20) at baseline.

^d^ No negative event experience in 6 months before initial assessment and with depressive symptoms (BDI ≥ 20) at baseline.

^e^ Adjusted for age, study center, education, income, marital status, comorbidity, menopausal status (in women only), alcohol consumption, cigarette smoking, and physical activity.

^f^ Standardized coefficient.

^g^ Summation of item 1–5 from Patient Health Questionnaire‐9.

^h^ Summation of item 6–9 from Patient Health Questionnaire‐9.

## 4 DISCUSSION

Compared with relative “Resilient” group at baseline, we observed a significant decrease in the follow‐up CD‐RISC score in the “Reactive” depression group. People who exhibited reactive depression, that is, reporting past adverse events with consequent depressed symptoms, showed significantly reduced scores overall and on most of the subfactors of CD‐RISC, indicating poor resilience status at follow‐up. This pattern did not differ by sex, which is in line with the previous validation study of the CD‐RISC in Korea (Jung et al., 2012).

It is intriguing that in our results, people who had reactive depression tended to exhibit fewer resilience traits after five years. Several previous studies explored the determining factors of resilience, ranging from socio‐environmental factors, cognitive‐behavioral patterns, and genetics to physical status (Choi et al., 2019). However, few studies have sought to explain the more significant reduction of the resilience trait after depression, especially in people who had depressive symptoms as a reaction to a stressful life event. It is possible that people who suffered depression at baseline already showed weak resilience traits, a result lasting over five years. It is also likely that adverse life events would reduce the resilience score; however, people manifesting the relative resilience were protected from further resilience impairment.

When comparing the baseline age of groups with resilience status, it is interesting to mention that relatively included younger participants were included in the “Resilient” group. However, other studies are suggesting the opposite direction. In young children, resilience factors, including self‐esteem, are known to grow as age increases (Sun & Stewart, 2007). In a study comparing two age groups (26 years or under vs. 65 years or older), the older adults were more resilient, including emotional regulation and problem‐solving (Gooding et al., 2012). However, our study indicates that people after midlife, certain factors, including general health status or social engagement, which people forfeit as the age increased, would reduce the psychological resilience trait.

To note, we did not observe a significant relationship between baseline relative resilience status and HRV indices at follow‐up (Table 3). In contrast, certain indices such as LF or LF/HF of HRV showed significant positive cross‐sectional associations with CD‐RISC scores. (Table S2). The authors who originally developed the CD‐RISC scale argue that the level might reflect the biology of resilience, which may capture changes in catecholaminergic activity and predict the efficacy of antidepressants (Connor & Davidson, 2003). Since HRV is a marker assessing the physiological domain of the sympathetic and parasympathetic influences of the autonomic nervous system, it is not surprising that the CD‐RISC score and individual indices of HRV exhibit significant correlations when measured simultaneously. In comparisons of baseline relative resilience with later CD‐RISC and HRV, CD‐RISC could serve to capture the remaining effect of a prior resilience process. In contrast, HRV seems to reflect a more instant state of resilience. In other words, CD‐RISC, rather than HRV, could partially capture the conversion of relative resilience to trait resilience. In previous HRV studies, people with post‐traumatic stress disorder, anxiety, and hyperarousal showed a reduced level of both HF and LF, indicating a chronic state of impaired parasympathetic inhibition. In this study, a higher resilience state, which could be reflected in a better CD‐RISC score, was positively correlated with LF/HF in men and with LF in women. HRV was shown to be predictive for a variety of clinical adverse outcomes such as mortality (Tsuji et al., 1994) and myocardial infarction (Buccelletti et al., 2009), and our study needs further follow‐up to evaluate the role of HRV in predicting other health outcomes.

The relative resilience state defined at baseline showed a significant association with later loneliness and depressive symptoms. People categorized as “Reactive” depression at baseline also exhibited increased depressive scores at follow‐up, with stronger associations with somatic‐affective factor scores. The two‐factor structure of depressive symptoms was frequently repeated in several studies, (De Jonge et al., 2007; Smolderen et al., 2009), which was divided into “cognitive affective” and “somatic‐affective” factors, and predominant proportion exhibited somatic‐affective symptoms in South Korea (Nam et al., 2011). The somatic‐affective subfactor is related with higher suicide rate (Lee et al., 2020) and physical symptoms such as arterial stiffness (Jeon et al., 2020). In this context, it is important to beware of the people who undergo depressive symptom after certain negative life event, preventing upcoming adverse physical events.

Regarding our radar chart plotting the five sub‐factor scores of CD‐RISC with baseline resilience status in both sexes, we hypothesize that previous depression categories could affect later components of resilience traits differentially by sex. For example, only men in the “Reactive” gave a relatively higher score for “resource to overcome adversity.” In contrast, only women in the Vulnerable group showed higher scores for self‐direction. However, both men and women in the “Resilient” group at baseline assessment showed relatively similar score distributions, with the highest scores overall for all five CD‐RISC sub‐scores, at follow‐up.

So far as we know, this is the most extensive longitudinal study comparing operational relative resilience status at baseline and follow‐up measurements on the CD‐RISC, HRV, and other mental health indices. Our sample was large enough to permit subgroup analysis by CD‐RISC sub‐factors, and it was also possible to categorize the baseline population into four groups, taking resilience as well as characteristics of depression into account. Since the data for this study were drawn from a large cohort study, we could obtain a variety of information for modeling. Our results did not compare measures directly and considered multiple confounders and potential mediators. The follow‐up period was similar for each participant in this study, five years, which enabled us to interpret the results more intuitively.

However, there are several limitations to this study. The interpretation of the results of the subfactors of CD‐RISC that we applied in this study needs caution since the factor structure and factor loadings have not been consistently replicated in other populations (Jung et al., 2012). The original factor structure also showed five sub‐factors, but the items contained in each factor differed from those of our analysis (Connor & Davidson, 2003), which may reflect the characteristics of the samples and sample recruitment of each study. Although a large number of variables was included in the final model, we could not obtain information such as family history or personal history of psychiatric diseases, use of psychopharmacological treatment for depression or any other psychiatric illness, adverse childhood experience, or adverse events during the follow‐up, which could be considered residual confounders. Potential measurement bias in measuring HRV and CD‐RISC could also underestimate the results. For example, as we spent 5 min for the HRV measurement in each person, however, recordings must have been done for longer times (e.g., 10–15 min) for the better reliability. This analysis only assessed relative resilience and trait resilience. However, further studies should also evaluate resilience as capacity and process with more detailed approach.

In summary, we observed a significant positive association between baseline relative resilience/risk categories and CD‐RISC at 5‐year follow‐up, but no significant association was observed with HRV. The trait resilience subfactor structure follow‐up was differently distributed by the baseline relative resilience and sex. The baseline relative resilience and risk categories were well correlated with subsequent mental health indices, such as depression and loneliness. However, research on this topic could be varied to take the cultural aspects of different people, societies, and economic situations into account. Our results need further replication with different samples. Additionally, validation of the CD‐RISC and our operational definition of resilience using other biomarkers such as specific neurohormonal transmitters or markers related to the hypothalamic‐pituitary axis, renin‐angiotensin system, insulin/growth hormone pathway, and immunity system is warranted.

## CONFLICTS OF INTEREST

None.

## AUTHOR CONTRIBUTIONS

Study design and concept: Jung Acquisition, access, analysis, or interpretation of data: Jung, Jeon Drafting of the manuscript: Jung Critical revision of the manuscript for important intellectual content: Jung, Jeon, Choi, Yang, Chae, Koenen, Kim Statistical analysis: Jung, Yang, Jeon Obtained funding: Jung Administrative, technical, or material support: Yang, Jeon Study supervision: Jung.

### Peer Review

The peer review history for this article is available at https://publons.com/publon/10.1002/brb3.2091.

## Supporting information

Table S1‐S2

## Data Availability

The data that support the findings of this study are available from the corresponding author upon reasonable request.
