# Supplementary material for: Correlates of psychological resilience and risk: Prospective associations of self‐reported and relative resilience with Connor‐Davidson resilience scale, heart rate variability, and mental health indices
Source: Brain Behav. 2021 Feb 27;11(5):e02091. doi: 10.1002/brb3.2091 (PMC8119814; doi:10.1002/brb3.2091)
Supplement: Supplementary file 1 — Table S1‐S2 [file BRB3-11-e02091-s001.docx]

Supplementary Table 1. Comparison of included (N=500) and excluded (N=307) participants among those registered in the 1^st^ year of CMERC baseline study

| Participants' Characteristics | Total (N=807) | | Included in follow-up study (N=500) | | Excluded in follow-up study (N=307) | | p-value |
| --- | --- | --- | --- | --- | --- | --- | --- |
|  |  |  |  |  |  |  |  |
| Age, Mean (SD) | 50.9 | (9.0) | 51.8 | (8.4) | 49.4 | (9.6) | <.001 |
| Sex |  |  |  |  |  |  |  |
| Male, N (%) | 236 | (29.2) | 151 | (30.2) | 85 | (27.7) | 0.446 |
| Female, N (%) | 571 | (70.8) | 349 | (69.8) | 222 | (72.3) |  |
| Socio-economic variables |  |  |  |  |  |  |  |
| Education: High school or more, N (%) | 322 | (39.9) | 197 | (39.4) | 125 | (40.7) | 0.711 |
| Highest quartile of Household income/year, N (%) | 153 | (19.0) | 88 | (17.6) | 65 | (21.2) | 0.209 |
| Currently married, living together, N (%) | 695 | (86.1) | 435 | (87.0) | 260 | (84.7) | 0.357 |
| Presence of major comorbidity, N (%) | 359 | (44.5) | 237 | (47.4) | 122 | (39.7) | 0.034 |
| Hypertension, N (%) | 190 | (23.5) | 112 | (22.4) | 78 | (25.4) | 0.328 |
| Diabetes, N (%) | 53 | (6.6) | 30 | (6.0) | 23 | (7.5) | 0.406 |
| Body Mass Index(kg/m^2^), Mean (SD) | 23.7 | (3.0) | 23.7 | (2.9) | 23.8 | (3.2) | 0.976 |
| Lifestyle factors, N(%) |  |  |  |  |  |  |  |
| Current cigarette smoker | 100 | (12.4) | 50 | (10.0) | 50 | (16.3) | 0.009 |
| Current alcohol consumer | 495 | (61.3) | 301 | (60.2) | 194 | (63.2) | 0.397 |
| Regular exercise^1^ | 373 | (46.2) | 241 | (48.2) | 132 | (43.0) | 0.150 |
| Menopaused (women only, 571) | 359 | (62.9) | 233 | (66.8) | 126 | (56.8) | 0.016 |
| Resilience status |  |  |  |  |  |  |  |
| Unexplosed and well^2^ | 270 | (33.5) | 102 | (33.6) | 168 | (33.2) | 0.132 |
| Resilient^3^ | 442 | (54.8) | 162 | (56.0) | 280 | (52.8) |  |
| Reactive Depression^4^ | 82 | (10.2) | 40 | (8.4) | 42 | (13.0) |  |
| Vulnerable Depression^5^ | 13 | (1.6) | 3 | (2.0) | 10 | (1.0) |  |
| Psychiatric assessments |  |  |  |  |  |  |  |
| Beck Depression Inventory-II (range:0-63) | 10.3 | (7.4) | 9.9 | (7.2) | 11.0 | (7.7) | 0.028 |
| Mini Mental State Examination-DS(range: 0-30) | 27.1 | (1.9) | 27.3 | (1.8) | 26.7 | (2.1) | <.001 |
| ^1^Defined as having moderate-vigorous physical activity more than 150 minutes in a week in average. | | | | | | | |
| ^2^No negative event experience in 6 months and no current depressive symptoms (BDI<20) | | | | | | | |
| ^3^Experienced negative events in 6 months but no current depressive symptoms (BDI<20) | | | | | | | |
| ^4^Experienced negative events in 6 months with current depressive symptoms (BDI≥20) | | | | | | | |
| ^5^No negative event experience in 6 months and with current depressive symptoms (BDI≥20) | | | | | | | |

| Supplementary Table 2. Association between Connor-Davidson Resilience Score and Heart Rate Variability Indices at follow-up | | | | | | | | | | | | | | | | | | | | | | | | | | | |
| --- | --- | --- | --- | --- | --- | --- | --- | --- | --- | --- | --- | --- | --- | --- | --- | --- | --- | --- | --- | --- | --- | --- | --- | --- | --- | --- | --- |
| Heart Rate Variability Indices^1^ | Connor-Davidson Risk Score | | | | | | | | | | | | | | | | | | | | | | | | | | |
|  | Total score | | | | Factor 1: Driving force for achievement | | | | Factor 2: Adaptability to adversity/stressful situation | | | | Factor 3: Resource to overcome adversity | | | | Factor 4: Self-direction | | | | Factor 5: Conformity to destiny | | | | | | |
|  | Adj.-β^2^ | (SE) | p-value | Adj.-β^2^ | | (SE) | p-value | Adj.-β^2^ | | (SE) | p-value | Adj.-β^2^ | | (SE) | p-value | Adj.-β^2^ | | (SE) | p-value | | | Adj.-β^2^ | (SE) | | p-value | |  |
| Men (N=143) | | | | | | | | | | | | | | | | | | | | | | | | | | | |
| SDNN | -0.012 | (0.10) | 0.905 | 0.136 | | (0.30) | 0.646 | 0.003 | | (0.30) | 0.993 | -0.037 | | (0.34) | 0.916 | -0.530 | | (0.71) | 0.457 | | | 0.309 | (0.92) | | 0.737 | |  |
| TP | -1.100 | (5.94) | 0.853 | 2.098 | | (18.16) | 0.908 | 2.793 | | (18.41) | 0.880 | -3.997 | | (21.13) | 0.850 | -39.479 | | (43.54) | 0.366 | | | 30.247 | (56.37) | | 0.592 | |  |
| LF | 1.243 | (1.74) | 0.477 | 3.447 | | (5.32) | 0.518 | 6.194 | | (5.37) | 0.251 | 5.217 | | (6.18) | 0.400 | -13.802 | | (12.75) | 0.281 | | | 17.399 | (16.48) | | 0.293 | |  |
| HF | 0.477 | (1.29) | 0.712 | 2.859 | | (3.93) | 0.468 | 4.438 | | (3.97) | 0.266 | -2.513 | | (4.58) | 0.584 | -0.611 | | (9.47) | 0.949 | | | 2.064 | (12.24) | | 0.866 | |  |
| LF/HF | **0.052** | **(0.02)** | **0.021** | 0.135 | | (0.07) | 0.050 | **0.139** | | **(0.07)** | **0.046** | **0.201** | | **(0.08)** | **0.012** | 0.076 | | (0.17) | 0.650 | | | 0.413 | (0.21) | | 0.054 | |  |
| RMSSD | 0.003 | (0.11) | 0.979 | 0.222 | | (0.35) | 0.527 | 0.140 | | (0.36) | 0.693 | -0.146 | | (0.41) | 0.722 | -0.202 | | (0.84) | 0.811 | | | -0.604 | (1.09) | | 0.580 | |  |
|  |  |  |  |  | |  |  |  | |  |  |  | |  |  |  | |  |  | | |  |  | |  | |  |
| Women (N=334) | | | | | | | | | | | | | | | | | | | |  |  | | |  | |  | |
| SDNN | 0.085 | (0.05) | 0.109 | **0.335** | | **(0.16)** | **0.036** | 0.148 | | (0.17) | 0.395 | 0.332 | | (0.20) | 0.105 | 0.317 | | (0.38) | 0.409 | | | 0.777 | (0.48) | | 0.107 | |  |
| TP | 7.421 | (5.17) | 0.152 | 27.365 | | (15.51) | 0.079 | 21.385 | | (16.92) | 0.207 | 26.400 | | (19.91) | 0.186 | 25.814 | | (37.37) | 0.490 | | | 12.740 | (47.04) | | 0.787 | |  |
| LF | **1.706** | **(0.76)** | **0.026** | **5.752** | | **(2.28)** | **0.012** | 4.659 | | (2.49) | 0.063 | **5.924** | | **(2.93)** | **0.044** | 7.739 | | (5.51) | 0.161 | | | 8.014 | (6.94) | | 0.249 | |  |
| HF | 1.160 | (1.11) | 0.299 | 5.068 | | (3.35) | 0.131 | 0.125 | | (3.65) | 0.973 | 3.728 | | (4.30) | 0.386 | 3.986 | | (8.05) | 0.621 | | | **20.971** | **(10.07)** | | **0.038** | |  |
| LF/HF | 0.001 | (0.01) | 0.854 | 0.002 | | (0.02) | 0.936 | -0.001 | | (0.02) | 0.957 | -0.014 | | (0.02) | 0.571 | 0.021 | | (0.05) | 0.656 | | | 0.037 | (0.06) | | 0.529 | |  |
| RMSSD | 0.036 | (0.07) | 0.588 | 0.254 | | (0.20) | 0.204 | -0.070 | | (0.22) | 0.749 | 0.093 | | (0.26) | 0.716 | -0.017 | | (0.48) | 0.972 | | | 0.934 | (0.60) | | 0.121 | |  |
| 1 SDNN=Standard Deviation of the NN interval; Psi=Physical Stress Index or Pressure Index; TP=Total power; LF=Low frequency; HF=High frequency; RMSSD=Square root of the mean of the sum of the square of differences between adjacent NN intervals; | | | | | | | | | | | | | | | | | | | | | | | | | | | |
| 2 Adjusted for age, comorbidity, physical exercise, and menopausal status (in women only) | | | | | | | | | | | | | | | | | | | | | | | | | | | |
